# Case Report: Ciclosporin A for Refractory Multisystem Inflammatory Syndrome in Children

**DOI:** 10.3389/fped.2022.890755

**Published:** 2022-05-31

**Authors:** Takayuki Suzuki, Tomohiro Suenaga, Aiko Sakai, Masaya Sugiyama, Masashi Mizokami, Ayumi Mizukami, Satoshi Takasago, Hiromichi Hamada, Nobuyuki Kakimoto, Takashi Takeuchi, Mina Ueda, Yuki Komori, Daisuke Tokuhara, Hiroyuki Suzuki

**Affiliations:** ^1^Department of Pediatrics, Wakayama Medical University, Wakayama, Japan; ^2^Genome Medical Sciences Project, Research Institute, National Center for Global Health and Medicine, Tokyo, Japan; ^3^Department of Pediatrics, Center Hospital of the National Center for Global Health and Medicine, Tokyo, Japan; ^4^Department of Pediatrics, Graduate School of Medicine, Chiba University, Chiba, Japan; ^5^Department of Pediatrics, Wakayama Rousai Hospital, Wakayama, Japan; ^6^Department of Pediatrics, Tsukushi Medical and Welfare Center, Iwade, Japan

**Keywords:** MIS-C, cytokine, ciclosporin A, Kawasaki disease, treatment option, phlebitis

## Abstract

Multisystem inflammatory syndrome in children (MIS-C) is a new syndrome involving the development of severe dysfunction in multiple organs after severe acute respiratory syndrome-coronavirus-2 (SARS-CoV-2) infection. Because the pathophysiology of MIS-C remains unclear, a treatment strategy has not yet been established. We experienced a 12-year-old boy who developed MIS-C at 56 days after SARS-CoV-2 infection and for whom ciclosporin A (CsA) was effective as a third-line treatment. He had a high fever on day 1, and developed a rash on the trunk, swelling in the cervical region, and palmar erythema on day 2. On days 3, he developed conjunctivitis and lip redness, and fulfilled the criteria for classical Kawasaki disease (KD). Although intravenous immunoglobulin infusion (IVIG) was started on day 4, fever persisted and respiratory distress and severe abdominal pain developed. On day 5, because he fulfilled the criteria for MIS-C, methylprednisolone pulse was started for 3 days as a second-line treatment. However, he did not exhibit defervescence and the symptoms continued. Therefore, we selected CsA as a third-line treatment. CsA was so effective that he became defervescent and his symptoms disappeared. In order to clarify the relationship with treatment and the change of clinical conditions, we examined the kinetics of 71 serum cytokines to determine their relationships with his clinical course during the three successive treatments. We found that CsA suppressed macrophage-activating cytokines such as, IL-12(p40), and IL-18 with improvement of his clinical symptoms. CsA may be a useful option for additional treatment of patients with MIS-C refractory to IVIG + methylprednisolone pulse.

## Introduction

Multisystem inflammatory syndrome in children (MIS-C) is a new syndrome that was first reported in Europe and the United States in 2020. Although the mechanism for onset of MIS-C remains unclear, recent manuscripts have suggested potential mechanisms from the viewpoint of various immunological angles (1–6). Based on the cytokine kinetics in MIS-C, a wide variety of cells related to both innate immunity and adaptive immunity, such as macrophages, neutrophils, T cells, and B cells, may be activated (4–6).

Regarding treatment of MIS-C, no definite treatment has been established to date. However, based on the similarity with KD vasculitis in many clinical aspects, intravenous immunoglobulin infusion (IVIG) alone or IVIG plus steroid are recommended as the first line treatment (7). If patients with MIS-C are refractory to the first-line treatment, biological agents such as infliximab (IFX; anti-tumor necrosis factor-α antagonist) (8), anakinra (interleukin [IL]-1 blocker) (9, 10), and tocilizumab (IL-6 receptor inhibitor) (11) are recommended as a second-line treatment. However, these options are also not definitively established.

Here, we report a patient with MIS-C in whom ciclosporin A (CsA) was effective as a third-line treatment. We examined the kinetics of 71 serum cytokines and gave discussion to determine their changes in accordance with the treatments and clinical symptoms during the patient’s clinical course.

## Case Report

A 12-year-old boy with no particular history of medical problems was diagnosed with severe acute respiratory syndrome-coronavirus-2 (SARS-CoV-2) infection by polymerase chain reaction (PCR) analysis of a nasopharyngeal swab sample at 56 days before onset of MIS-C. He was admitted to his previous hospital because of low-grade fever and sore throat. His father was Caucasian, and his mother was Japanese.

At 56 days after the diagnosis of SARS-CoV-2 infection, he developed a fever (39.8°C) on day 1. On the next day (day 2), he had a rash on his trunk, swelling and pain in both sides of the cervical region, and palmar erythema. He was therefore re-admitted to the previous hospital, and ceftriaxone (50 mg/kg/day) was initiated. On day 3, he developed conjunctivitis and lip redness. On day 4, his fever persisted, and because he fulfilled the criteria for classical KD. Therefore, IVIG (2 g/kg) and aspirin (30 mg/kg) were started. However, the fever persisted, and abdominal pain and chest discomfort developed. On day 5, he was transferred to our hospital.

On admission, he had a fever of 41.0°C, severe pain throughout the abdomen, and bilateral cervical lymph node swelling with pain. Physical examination revealed multilobular cervical lymphadenopathy with a maximum diameter of 20 mm, and obvious redness of the lips and oral cavity. There were significant bilateral bulbar conjunctival congestions, and rashes on the face, trunk, and distal extremities. Although his blood pressure was 99/37 mmHg and within the reference range, he showed respiratory distress, including heart rate of 142 beats/min, respiratory rate of 50 breaths/min, and SpO_2_ of 99% (nasal O_2_: 2 L/min). Furthermore, cardiomegaly (cardiothoracic ratio: CTR; 54.6%) and pulmonary congestion were seen on both chest X-ray and computed tomography examinations, and intestinal gas showed prominent expansion on an abdominal X-ray (Supplementary Figures 1A,B). Electrocardiogram showed sinus tachycardia (heart rate: 142 beats/min), with flat and negative T waves on the V5 and V6 leads, suggesting myocardial damage (Supplementary Figure 1C). Although he had a negative test result for SARS-CoV-2, based on a TRC Ready (Tosoh Bioscience, Tokyo, Japan) analysis of a nasopharyngeal swab, SARS-CoV-2 Antibody Detection Kit (IgG/IgM) (Kurabo Industries Ltd., Osaka, Japan) was positive and negative, respectively.

**FIGURE 1:**
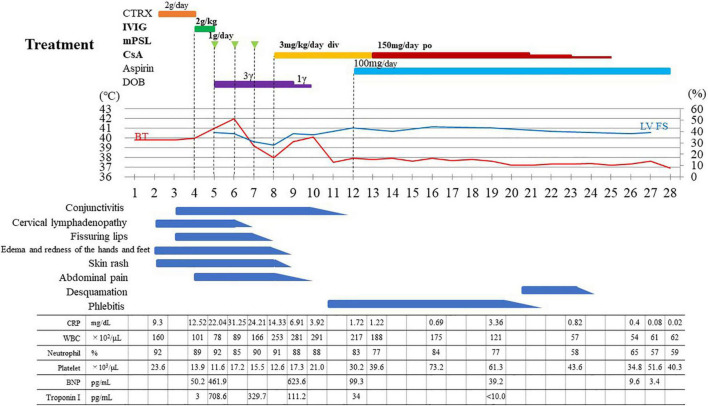
Treatment and clinical course of the patient. CTRX, ceftriaxone; IVIG, intravenous immunoglobulin infusion; mPSL, methylprednisolone pulse; CsA, ciclosporin A; DOB, dobutamine; LV FS, left ventricular fractional shortening; BT, body temperature; CRP, C-reactive protein (reference range < 0.14); WBC, white blood cell (reference range 4,000–10,700); BNP, B-type natriuretic peptide (reference range < 18.4); Toroponin I (reference range < 26.2); CTR, cardiothoracic ratio.

Laboratory data on admission are shown in Table 1. Elevated neutrophil count (7.137 × 10^3^/μL) and percentage of leukocytes (90%), reduced lymphocyte count (469/μL) and platelet count (116 × 10^3^/μL), and elevated C-reactive protein level (22.04 mg/dL) were noted. In addition, elevated soluble IL-2 receptor level (7,044 U/mL, reference range 122–496 U/mL), normal ferritin level (173 ng/mL, reference range 13–277 ng/mL), elevated fibrin degradation product level (36.2 μg/mL, reference range 0–5 μg/mL), elevated brain natriuretic hormone level (461.9 pg/mL, reference range < 18.4 pg/mL), and elevated troponin I (708.6 pg/mL, reference range < 26.2 pg/mL) were found. The urinalysis did not show pyuria. Based on the clinical symptoms and laboratory data, he was diagnosed with MIS-C because he fulfilled the World Health Organization (WHO), Centers for Disease Control and Prevention (CDC) and Royal College of Paediatrics and Child Health (RCPCH) criteria (12–14). His clinical disease course following hospitalization is shown in Figure 1. We selected a methylprednisolone pulse (mPSL; 25 mg/kg/day) as a second-line treatment according to the guideline (7–9). Anticoagulation therapy (heparin: 10 IU/kg/h) was administered during the methylprednisolone pulse administration. Dobutamine was started simultaneously for the signs of cardiac failure such as cardiomegaly and pulmonary edema. Although his fever increased and decreased with the three consecutive mPSL pulses, he did not exhibit defervescence. In addition, his KD-like symptoms, decreased cardiac function, and abdominal pain persisted. Most guidelines recommend a biological agent as a second-line and/or third-line treatment after IVIG and steroid treatment. However, we discontinued glucocorticoid therapy and selected CsA as the third-line treatment because his clinical symptoms also fulfilled the six principal diagnostic criteria for classical KD (15–17). Initially, CsA was started by continuous intravenous injection (3 mg/kg/day) because he had severe abdominal pain at that time (day 8). The route of CsA treatment was changed from continuous intravenous injection to oral administration (3.75 mg/kg/day, divided by 2) on day 13 when his main symptoms, decreased cardiac function, and abdominal pain disappeared. He became defervescent and his symptoms, such as severe pain, disappeared within several days starting CsA treatment. We confirmed that no CAAs developed by both repeated transthoracic echocardiography and 3D computed tomography during his clinical course (Supplementary Figure 2). He was discharged after improvement of symptoms on day 28, and he has been doing well since discharge without any complications. Therefore, we judged CsA to be clearly effective for this patient with MIS-C refractory to IVIG + mPSL. Although various symptoms and signs disappeared after CsA treatment, his peripheral limbs (both hands and feet) turned purple and severe pain occurred when they hung below the heart, such as in the standing and/or sitting position, on day 11. There were no significant findings such as thrombus on vascular echography and magnetic resonance imaging or right heart failure. We judged these phenomena to be venous stasis, because the pain and color changes to the skin rapidly improved on elevation of the peripheral limbs. Elastic bandage use improved these symptoms, and thus exercise therapy was performed with an elastic bandage. Under exercise rehabilitation therapy, the symptoms improved in 10 days (18).

**TABLE 1:** Laboratory data on admission.

			(Reference range)				(Reference range)				(Reference range)
WBC	7930	/μL	(4,000–10,700)	Na	131	mEq/L	(138–144)	CRP	22.04	mg/dL	(<0.14)
Neutrophil	90.0	%		K	3.3	mEq/L	(3.6–4.7)	PCT	23.20	ng/mL	(<0.05)
Eosinophil	1.0	%		Cl	100	mEq/L	(102–109)	sIL-2r	7,044	U/mL	(122–496)
Basophil	1.0	%		Ca	9.4	mg/dL	(8.7–10.1)	Ferritin	173	ng/mL	(13–277)
Monocyte	1.0	%		Cr	0.62	mg/dL	(0.39–0.62)	U-β_2_MG	125,826	μg/L	(<230)
Lymphocyte	6.0	%		UA	5.4	mg/dL	(3.0–7.0)				
RBC	412 × 10^4^	/μL	(415–540 × 10^4^)	BUN	23.3	mg/dL	(6.8–19.2)	BNP	461.9	pg/mL	(<18.4)
Hb	11.6	g/dL	(12.2–15.7)	TP	6.9	g/dL	(6.3–7.8)	TnI	708.6	pg/mL	(<26.2)
Ht	33.4	%	(35.8–45.0)	Alb	2.7	g/dL	(3.8–4.7)				
Plt	11.6 × 10^4^	/μL	(18.0–44.0 × 10^4^)	CK	61	IU/L	(51–270)	SARS-CoV-2	Antibody		
				CK-MB	<4	IU/L	(<12)		IgG (+)		
APTT	41.0	s	(25–35)	AST	48	IU/L	(15–31)		IgM (−)		
PTINR	1.42		(0.8–1.2)	ALT	32	IU/L	(9–32)				
Fib	511	mg/dL	(150–350)	LDH	283	IU/L	(145–270)				
FDP	32.6	μg/mL	(0–5)	TB	1.5	mg/dL	(0.3–1.1)				
				DB	0.8	mg/dL	(0.0–0.3)				

*WBC, white blood cells; RBC, red blood cells; Hb, hemoglobin; Ht, hematocrit; Plt, platelets; APTT, activated partial thromboplastin time; PTINR, prothrombin time-international normalized ratio; Fib, fibrinogen; FDP, fibrin degradation product; Cr, creatinine; UA, uric acid; BUN, blood urea nitrogen; TP, total protein; Alb, albumin; CK, creatine kinase phosphokinase; CK-MB, creatine kinase MB type; AST, aspartate transaminase; ALT, alanine aminotransferase; LDH, lactate dehydrogenase; TB, total bilirubin; DB, direct bilirubin; CRP, C-reactive protein; PCT, procalcitonin; sIL-2R, soluble interleukin-2 receptor; U-B2MG, urinary β2-microglobulin; BNP, brain natriuretic peptide; TnI, troponin I; SARS-CoV-2, severe acute respiratory syndrome-coronavirus-2.*

## Analysis of Cytokine Kinetics

**FIGURE 2:**
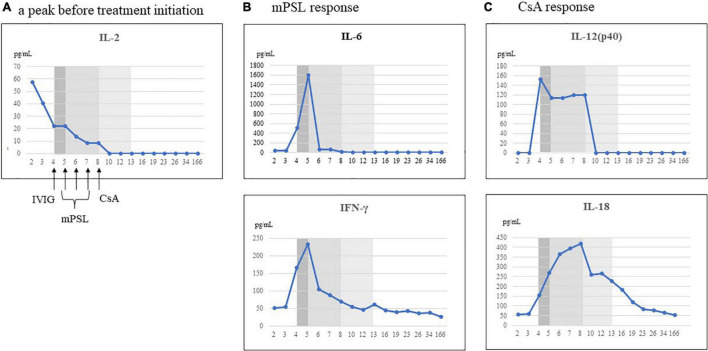
Representative cytokines that responded to each treatment. We classified the treatment response when the cytokine decreased by 50% from the peak levels, referenced to the day 166 levels for each cytokine. (A) Cytokine decreased prior to initiation of treatment. (B) Cytokines that responded to methylprednisolone (mPSL) treatment. (C) Cytokines that responded to ciclosporin A (CsA) treatment. IL, interleukin; IFN, interferon; IVIG, intravenous immunoglobulin infusion.

We evaluated his cytokine kinetics using archived serum samples collected intermittently from day 2 to day 166 of illness, in order to clarify the relationship with treatment and the change of clinical conditions. We performed comprehensive assays for cytokines using a BioPlex 3D system (Bio-Rad Laboratories Inc., Hercules, CA, United States) and BioPlex Human cytokine48/chemokine40 screening panel (Bio-Rad Laboratories Inc.). We also performed chemiluminescence enzyme immunoassay analyses using an HISCL-5000 system (Sysmex Asia Pacific Pte Ltd., Singapore) and ELISA assay (R&D Systems Inc., Minneapolis, MN, United States). Using these methods, we investigated the changes in 71 cytokines during the patient’s disease course. We classified a treatment response when a cytokine decreased by 50% from its peak level, referenced by the day 166 level for that cytokine. IL-2 and CXCL2 decreased before the initiation of treatment. Although no cytokines were completely suppressed by IVIG alone, proinflammatory cytokines (IL-6, IL-1β), interferon (IFN)-γ and IFN-γ-related chemokines (CXCL9, CXCL10, and CXCL11), and many chemokines (CCL1, CCL2, CCL8, CCL19, CCL20, CCL22, and CCL27) were strongly suppressed by mPSL (Table 2 and Figure 2). Although IL-6 was completely suppressed by mPSL, the patient did not become defervescent. As the third-line treatment, CsA mainly suppressed macrophage-activating cytokines such as IL-12(p40), and IL-18, and he became defervescent with disappearance of abdominal pain (Supplementary Figures 3-1, 3-2).

**TABLE 2:** Summary of cytokines which respond to each treatment (*n* = 71).

A peak before treatment initiation	mPSL response			CsA response			Unknown	
IL-2	IFN-γ	IL-6		IL-12 (p40)	IL-18		IL-4	CCL24
CXCL2	CXCL9	CXCL10	CXCL11	TNF-α	VEGF		IL-7	CXCL5
	CCL1	CCL2	CCL8	HGF			IL-9	CXCL6
	CCL19	CCL20					IL-12 (p70)	CXCL12
	IL-1β	CCL7	CXCL1	IL-1α	CCL23	LIF	IL-15	CXCL16
	IL-3	CCL17	G-CSF	IL-1ra	CCL25	GM-CSF	IL-16	FGF2
	IL-5	CCL21	IFN-λ3	IL-2Rα	CX3CL1	SCF	CCL3	M-CSF
	IL-8	CCL22		IL-13	CXCL13	NGF-β	CCL4	PDGF-BB
	IL-10	CCL26		IL-17	IFN-α2	IFN-λ2	CCL5	SCGF-b
		CCL27			MIF		CCL11	TNF-β
							CCL13	TRAIL
							CCL15	IFN-λ1

*We classified the treatment response when the cytokine decreased by 50% from the peak levels, referenced to the day 166 levels for each cytokine. mPSL, methylprednisolone; CsA, ciclosporin A; IL, interleukin; CXCL, C-X-C motif chemokine ligand; IFN, interferon; CCL, C-C motif ligand; G-CSF, granulocyte colony stimulating factor; TNF, tumor necrosis factor; VEGF, vascular endothelial growth factor; HGF, hepatocyte growth factor; ra, receptor antagonist; Rα, receptor α; LIF, leukemia inhibitory factor; MIF, macrophage migration inhibitory factor; GM-CSF, granulocyte macrophage colony stimulating factor; SCF, stem cell factor; NGF, nerve growth factor; FGF, fibroblast growth factor; M-CSF, macrophage colony stimulating factor; PDGF, platelet derived growth factor; SCGF, stem cell growth factor; TRAIL, tumor necrosis factor related apoptosis-inducing ligand.*

## Discussion

We reported a patient with MIS-C in whom CsA was effective as a third-line treatment. We examined the kinetics of 71 serum cytokines during the patient’s clinical course. In the present case, three successive treatments (IVIG, mPSL, and CsA) were performed during the time course, and thus we could distinguish the relationship between each treatment and the cytokine changes. Few cytokines were suppressed by IVIG alone. However, IL-6, IFN-γ, and IFN-γ-induced chemokines (CXCL9, CXCL10, and CXCL11) (19) and chemokines (CCL1, CCL2, CCL8, CCL19, and CCL20) were strongly suppressed by mPSL. Although IL-6 was completely suppressed by mPSL, the patient did not become defervescent. Meanwhile, CsA suppressed cytokines related to innate immune responses such as IL-12(p40), IL-18, and growth factors such as vascular endothelial growth factor and hepatocyte growth factor. Thereafter, the patient became defervescent. In our previous study, we reported the kinetics of 71 cytokines in a 9-year-old girl with MIS-C during her entire clinical course (6). However, both IVIG and prednisolone were started simultaneously in that case, and we could not distinguish the relationship between each treatment and the cytokine changes. Nevertheless, after IVIG and prednisolone administration as the first-line treatment, IL-6, IL-10, IL-17, IL-8, and CCL20 rapidly decreased, and the patient became defervescent. Meanwhile, IFN-γ is an indicator of Th1 type immune responses, and IFN-γ-induced cytokines such as CXCL9, CXCL10 decreased gradually, rather than rapidly. There is a report that IFN-γ plays a central role in the pathophysiology of MIS-C, indicating that IFN-γ may be a notable cytokine for understanding the state of MIS-C patients (19, 20). Furthermore, the present case was considered to be a more severe case of MIS-C, with higher levels of IL-12(p40), IL-18, IFN-γ, and IFN-γ-induced chemokines such as CXCL9 and CXCL10 than in our previous report.

Because these cytokine kinetics were assessed for three separate treatments during the time course, the relationships between each of the treatments and the changes in cytokines seemed to be clear in this report. However, it is not definitive whether the cytokines kinetics tightly correspond to each treatment, and it remains possible that there may be synergetic effects with the prior treatment. Among the data, it is particularly interesting that the patient did not become defervescent after complete suppression of IL-6 by mPSL, and required a third-line treatment. Thus, it is possible that tocilizumab as an additional treatment may be an inappropriate choice, especially those who are refractory to previous steroid treatment.

MIS-C and KD are similar in many clinical presentations, and 25–65.5% of MIS-C patients also have symptoms that resemble classical KD (21). Because our case was not only definite MIS-C but also fulfilled the criteria for classical KD, we selected CsA as the third-line treatment when he showed resistance to both IVIG and mPSL (16, 17). We usually select oral administration for CsA, but chose continuous infusion (3 mg/kg/day) in the present patient between day 8 and day 13, because he had remarkable gastrointestinal symptoms such as severe pain. After his abdominal pain disappeared, we changed the administration route of CsA from infusion to per oral (150 mg/day, divided by 2). His plasma level of CsA was 321–372 ng/ml at infusion and his trough level was 87-97 ng/ml at oral administration. All symptoms and signs of MIS-C in our patient improved after CsA treatment and no CAAs developed.

Regarding the effect of CsA in the present case, it is possible that the suppressive effects of CsA on macrophages and dendritic cells may have contributed to the clinical improvements, because the levels of cytokines related to innate immune responses such as IL-12(p40) and IL-18 were decreased at defervescence.

Ciclosporin A was originally considered to suppress the immune system through inactivation of the nuclear factor of activated T-cells pathway. However, according to pathophysiology of MIS-C, both innate immunity and adaptive immunity are highly activated. If this hypothesis is true, the mechanism of action for CsA may not be sufficient to control the total immune activation in MIS-C. Therefore, CsA may not have been recommended as an additional option for treatment of MIS-C in the treatment guideline (7–9). However, recent studies have clarified that CsA may exert direct suppressive effects on not only T cells but also innate immune cells such as dendritic cells, macrophages, and neutrophils (22–24). Indeed, cytokines related to innate immunity were suppressed by CsA in the present patient with MIS-C. These findings suggest that CsA may be a useful option for MIS-C refractory to IVIG + mPSL, which is known to involve activation of both innate immunity and adaptive immunity.

Although we have no evidence to explain why our patient developed venous stasis, we propose one possibility. The changes in skin color of his limbs were not chilblain-like skin lesions associated with SARS-CoV-2 infection (25), because the changes developed depending on the vertical position of the limbs and disappeared immediately in the horizontal position. Thus, we judged these phenomena to be venous stasis. Therefore, vasculitis caused by MIS-C may be not only arteritis but also phlebitis. Several reports have described that autoantibodies to autoantigens expressed in endothelial and cardiac tissues may trigger onset of MIS-C (1, 4, 26). Thus, it is possible that vasculitis may develop regardless of involvement of an artery or a vein. Therefore, the function of venous valves may be insufficient.

There are several limitations in the present case report. This is the first report to discuss the possibility of CsA treatment for MIS-C refractory to IVIG + mPSL. In addition, it is possible that the effect of CsA as a third-line treatment may have involved the summed results for IVIG and mPSL. Further studies are warranted to evaluate CsA treatment in refractory MIS-C patients.

## Conclusion

The findings in the present case regarding the relationship between cytokine kinetics and treatment response suggest that CsA may be a useful option for patients with MIS-C who are refractory to IVIG + methylprednisolone pulse treatment.

## Data Availability Statement

The original contributions presented in the study are included in the article/Supplementary Material, further inquiries can be directed to the corresponding author.

## Ethics Statement

The studies involving human participants were reviewed and approved by the Ethics Committee of Wakayama Medical University (No: 3282) and National Center for Global Health and Medicine (NCGM-S-004245). Written informed consent was obtained from the patient and his parents for the publication of this case report. Written informed consent to participate in this study was provided by the participants’ legal guardian/next of kin.

## Author Contributions

TaS performed the literature review and wrote the first draft of the manuscript. MU, YK, ToS, NK, TT, and HS assisted in the treatment of the patient. AS, MS, MM, AM, and ST contributed to the analysis and interpretation of cytokines and assisted in the preparation of the manuscript. HH and DT contributed to critically review of the manuscript. All authors have approved the final manuscript and have agreed to be accountable for all aspects of the work.

## Conflict of Interest

The authors declare that the research was conducted in the absence of any commercial or financial relationships that could be construed as a potential conflict of interest.

## Publisher’s Note

All claims expressed in this article are solely those of the authors and do not necessarily represent those of their affiliated organizations, or those of the publisher, the editors and the reviewers. Any product that may be evaluated in this article, or claim that may be made by its manufacturer, is not guaranteed or endorsed by the publisher.

## Supplementary Material

Supplementary Figure 1 (A) Chest and abdominal X-ray image. Cardiothoracic ratio is 54.6%, pulmonary congestion is present, and intestinal gas shows prominent expansion. (B) Computed tomography image. Hair line is present. (C) Electrocardiogram. Heart rate is 142 beats/min, sinus rhythm is present, and T waves on the V5 and V6 leads are flat and negative, suggesting myocardial damage.

Supplementary Figure 2 3D-Computed Tomography (day 45).

Supplementary Figure 3-1 Cytokines which responded to ciclosporin A (CsA). IL, interleukin; ra, receptor antagonist; Rα, receptor α; CCL, C–C motif ligand; CX3CL, C-X3-C motif ligand; IFN, interferon.

Supplementary Figure 3-2 Cytokines which responded to ciclosporin A (CsA). TNF, tumor necrosis factor; LIF, leukemia inhibitory factor; HGF, hepatocyte growth factor; MIF, macrophage migration inhibitory factor; GM-CSF, granulocyte macrophage colony-stimulating factor; SCF, stem cell factor; VEGF, vascular endothelial growth factor; NGF, nerve growth factor; IFN, interferon.
