# Supplementary material for: Case Report: Ciclosporin A for Refractory Multisystem Inflammatory Syndrome in Children
Source: Front Pediatr. 2022 May 31;10:890755. doi: 10.3389/fped.2022.890755 (PMC9194446; doi:10.3389/fped.2022.890755)
Supplement: Supplementary Figure 1 — (A) Chest and abdominal X-ray image. Cardiothoracic ratio is 54.6%, pulmonary congestion is present, and intestinal gas shows prominent expansion. (B) Computed tomography image. Hair line is present. (C) Electrocardiogram. Heart rate is 142 beats/min, sinus rhythm is present, and T waves on the V5 and V6 leads are flat and negative, suggesting myocardial damage. [file Presentation_1.PPTX]

## Slide 1
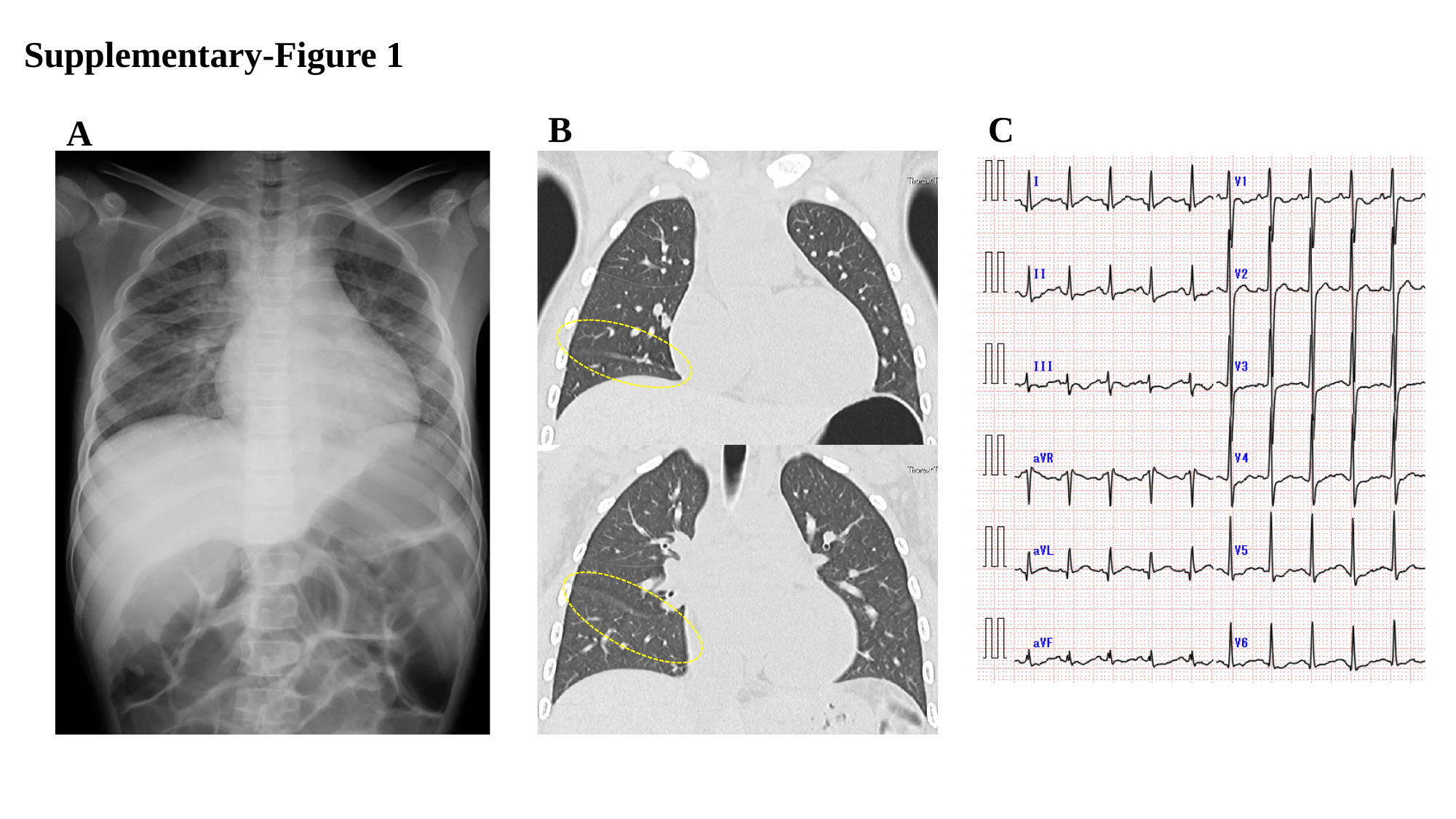

Supplementary-Figure 1
C
B
A

## Slide 2
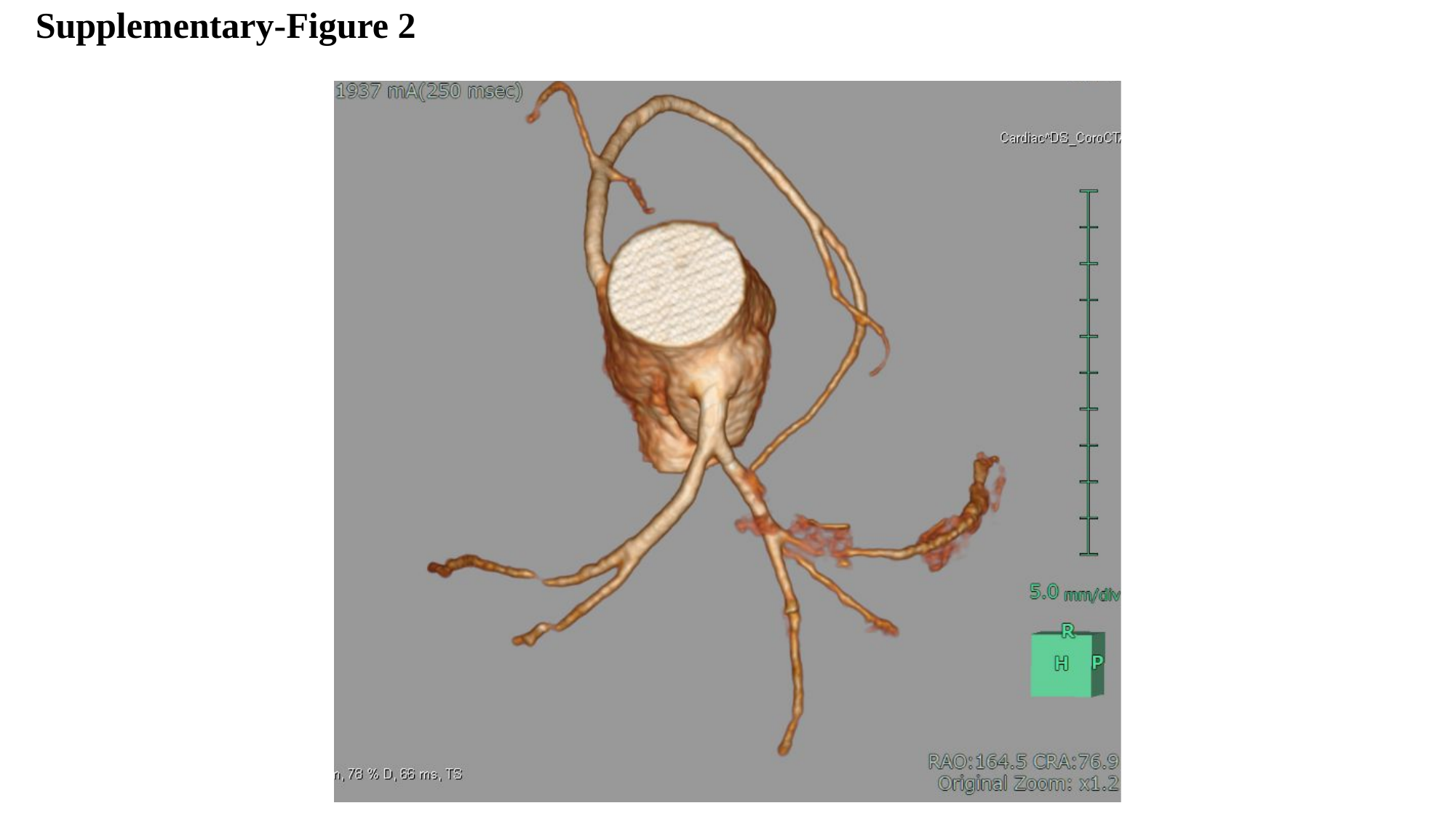

Supplementary-Figure 2

## Slide 3
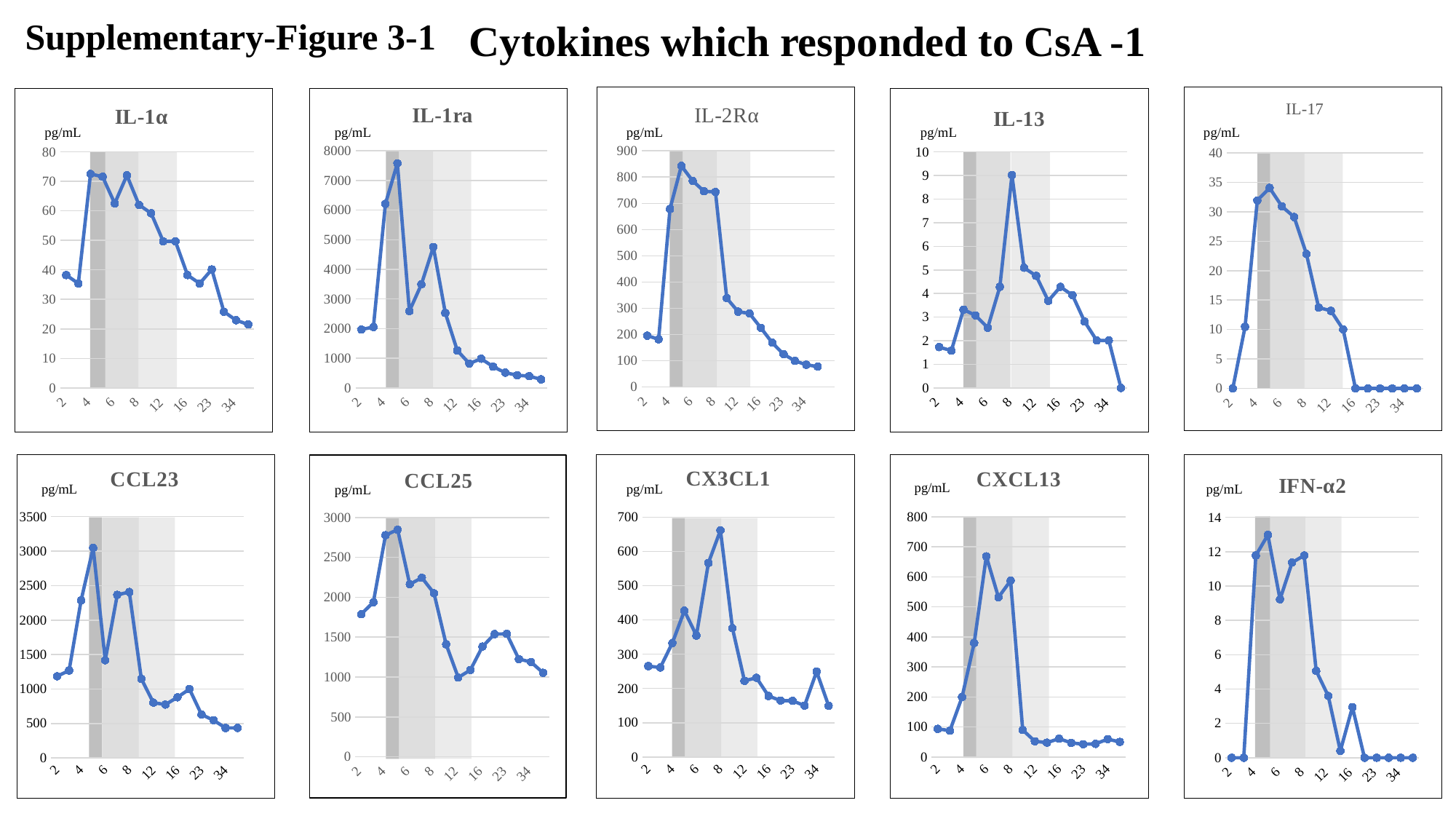

Supplementary-Figure 3-1
 Cytokines which responded to CsA -1
### Chart: IL-2Rα
| Category | Hu IL-2Ra (13) |
|---|---|
| 2 | 195.21 |
| 3 | 181.49 |
| 4 | 678.57 |
| 5 | 842.54 |
| 6 | 785.27 |
| 7 | 745.84 |
| 8 | 743.09 |
| 10 | 338.1 |
| 12 | 286.71 |
| 13 | 279.73 |
| 16 | 225.2 |
| 19 | 168.25 |
| 23 | 124.64 |
| 26 | 98.94 |
| 34 | 84.41 |
| 166 | 77.36 |
| | None |
### Chart: IL-17
| Category | Hu IL-17 (76) |
|---|---|
| 2 | 0.0 |
| 3 | 10.46 |
| 4 | 31.92 |
| 5 | 34.05 |
| 6 | 30.91 |
| 7 | 29.11 |
| 8 | 22.83 |
| 10 | 13.73 |
| 12 | 13.17 |
| 13 | 10.01 |
| 16 | 0.0 |
| 19 | 0.0 |
| 23 | 0.0 |
| 26 | 0.0 |
| 34 | 0.0 |
| 166 | 0.0 |
### Chart: IL-1α
| Category | Hu IL-1a (63) |
|---|---|
| 2 | 38.24 |
| 3 | 35.38 |
| 4 | 72.53 |
| 5 | 71.57 |
| 6 | 62.53 |
| 7 | 72.05 |
| 8 | 62.05 |
| 10 | 59.2 |
| 12 | 49.68 |
| 13 | 49.68 |
| 16 | 38.24 |
| 19 | 35.38 |
| 23 | 40.15 |
| 26 | 25.81 |
| 34 | 22.94 |
| 166 | 21.5 |
### Chart: IL-1ra
| Category | Hu IL-1ra (25) |
|---|---|
| 2 | 1968.61 |
| 3 | 2055.24 |
| 4 | 6208.77 |
| 5 | 7580.06 |
| 6 | 2590.36 |
| 7 | 3491.96 |
| 8 | 4756.64 |
| 10 | 2531.23 |
| 12 | 1263.09 |
| 13 | 824.35 |
| 16 | 983.42 |
| 19 | 714.95 |
| 23 | 514.65 |
| 26 | 426.61 |
| 34 | 394.79 |
| 166 | 288.1 |
### Chart: IL-13
| Category | Hu IL-13 (51) |
|---|---|
| 2 | 1.73 |
| 3 | 1.58 |
| 4 | 3.32 |
| 5 | 3.07 |
| 6 | 2.55 |
| 7 | 4.28 |
| 8 | 9.02 |
| 10 | 5.09 |
| 12 | 4.75 |
| 13 | 3.69 |
| 16 | 4.28 |
| 19 | 3.93 |
| 23 | 2.81 |
| 26 | 2.01 |
| 34 | 2.01 |
| 166 | 0.0 |pg/mL
pg/mL
pg/mL
pg/mL
pg/mL
### Chart: CCL23
| Category | Hu MPIF-1/CCL23 (37) |
|---|---|
| 2 | 1184.53 |
| 3 | 1268.86 |
| 4 | 2288.11 |
| 5 | 3047.5 |
| 6 | 1417.39 |
| 7 | 2366.53 |
| 8 | 2407.94 |
| 10 | 1148.08 |
| 12 | 802.97 |
| 13 | 773.99 |
| 16 | 879.98 |
| 19 | 999.46 |
| 23 | 629.43 |
| 26 | 547.06 |
| 34 | 435.49 |
| 166 | 436.1 |
### Chart: CCL25
| Category | Hu TECK/CCL25 (46) |
|---|---|
| 2 | 1788.28 |
| 3 | 1938.23 |
| 4 | 2778.75 |
| 5 | 2848.12 |
| 6 | 2163.38 |
| 7 | 2244.78 |
| 8 | 2050.76 |
| 10 | 1410.39 |
| 12 | 993.88 |
| 13 | 1088.05 |
| 16 | 1382.26 |
| 19 | 1538.46 |
| 23 | 1541.58 |
| 26 | 1225.9 |
| 34 | 1188.33 |
| 166 | 1053.54 |
### Chart: CX3CL1
| Category | Hu Fractalkine/CX3CL1 (77) |
|---|---|
| 2 | 265.08 |
| 3 | 261.24 |
| 4 | 332.82 |
| 5 | 426.99 |
| 6 | 354.52 |
| 7 | 566.58 |
| 8 | 661.88 |
| 10 | 376.19 |
| 12 | 222.28 |
| 13 | 231.28 |
| 16 | 177.99 |
| 19 | 164.69 |
| 23 | 163.8 |
| 26 | 150.01 |
| 34 | 249.33 |
| 166 | 149.36 |
### Chart: CXCL13
| Category | Hu BCA-1/CXCL13 (74) |
|---|---|
| 2 | 93.67 |
| 3 | 87.65 |
| 4 | 199.37 |
| 5 | 379.61 |
| 6 | 668.17 |
| 7 | 531.84 |
| 8 | 587.28 |
| 10 | 90.04 |
| 12 | 52.31 |
| 13 | 47.67 |
| 16 | 61.01 |
| 19 | 46.82 |
| 23 | 42.63 |
| 26 | 43.57 |
| 34 | 59.46 |
| 166 | 50.22 |
### Chart: IFN-α2
| Category | |
|---|---|
| 2 | 0.0 |
| 3 | 0.0 |
| 4 | 11.78 |
| 5 | 12.98 |
| 6 | 9.23 |
| 7 | 11.37 |
| 8 | 11.78 |
| 10 | 5.06 |
| 12 | 3.6 |
| 13 | 0.4 |
| 16 | 2.95 |
| 19 | 0.0 |
| 23 | 0.0 |
| 26 | 0.0 |
| 34 | 0.0 |
| 166 | 0.0 |pg/mL
pg/mL
pg/mL

## Slide 4
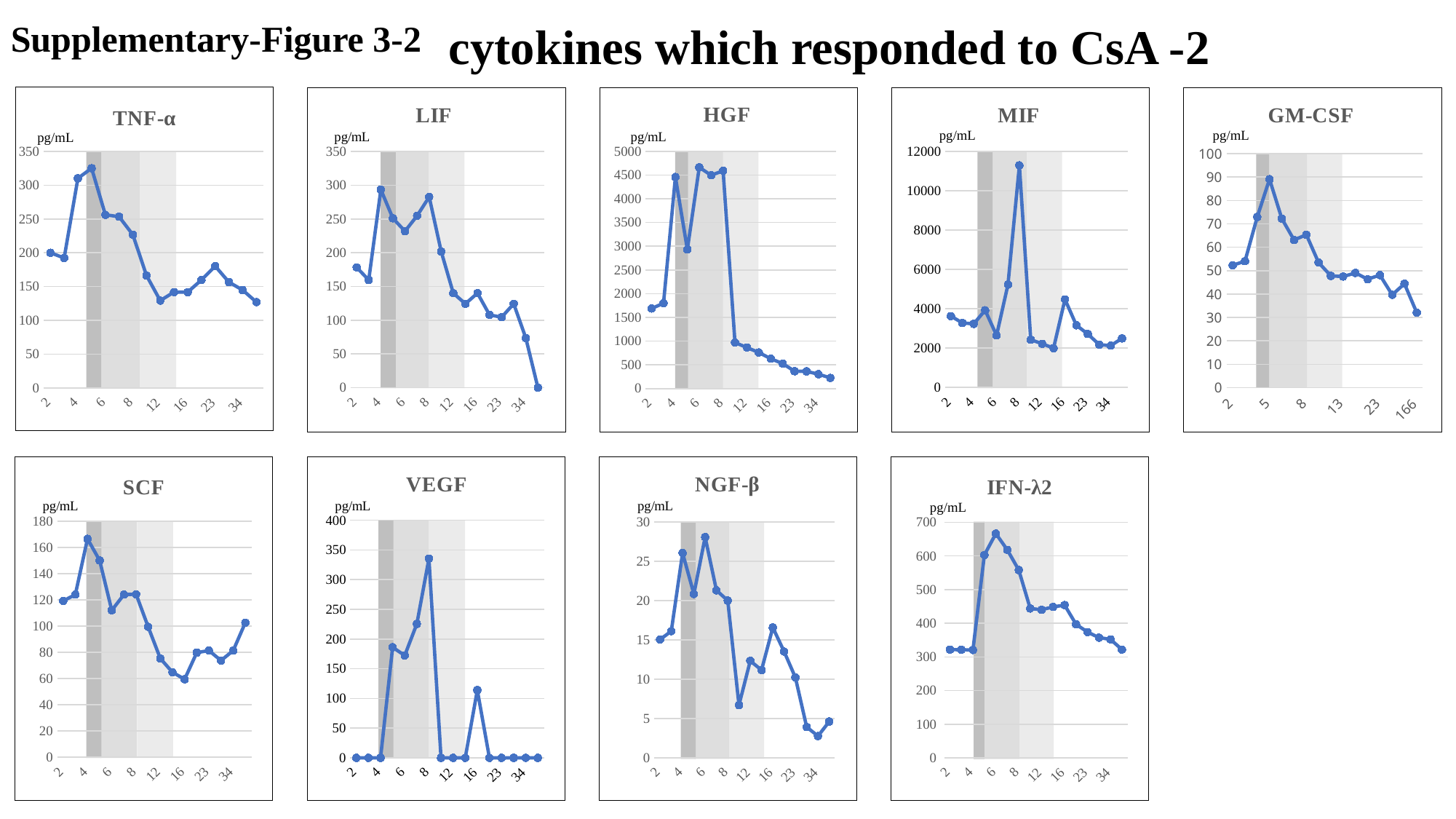

Supplementary-Figure 3-2
 cytokines which responded to CsA -2
### Chart: TNF-α
| Category | Hu TNF-a (36) |
|---|---|
| 2 | 200.02 |
| 3 | 192.28 |
| 4 | 310.08 |
| 5 | 325.06 |
| 6 | 256.22 |
| 7 | 253.38 |
| 8 | 226.74 |
| 10 | 166.27 |
| 12 | 129.25 |
| 13 | 141.79 |
| 16 | 141.79 |
| 19 | 159.75 |
| 23 | 180.2 |
| 26 | 156.58 |
| 34 | 144.81 |
| 166 | 127.01 |
### Chart: LIF
| Category | Hu LIF (29) |
|---|---|
| 2 | 178.32 |
| 3 | 159.6 |
| 4 | 293.57 |
| 5 | 251.07 |
| 6 | 232.07 |
| 7 | 254.81 |
| 8 | 282.87 |
| 10 | 201.79 |
| 12 | 140.05 |
| 13 | 124.31 |
| 16 | 140.05 |
| 19 | 107.82 |
| 23 | 104.41 |
| 26 | 124.31 |
| 34 | 73.5 |
| 166 | 0.0 |
### Chart: HGF
| Category | Hu HGF (62) |
|---|---|
| 2 | 1685.85 |
| 3 | 1797.47 |
| 4 | 4457.66 |
| 5 | 2930.5 |
| 6 | 4662.7 |
| 7 | 4495.62 |
| 8 | 4589.84 |
| 10 | 970.22 |
| 12 | 864.13 |
| 13 | 759.75 |
| 16 | 628.01 |
| 19 | 525.55 |
| 23 | 365.29 |
| 26 | 361.48 |
| 34 | 302.8 |
| 166 | 223.6 |
### Chart: MIF
| Category | Hu MIF (35) |
|---|---|
| 2 | 3617.37 |
| 3 | 3273.48 |
| 4 | 3220.08 |
| 5 | 3914.91 |
| 6 | 2634.55 |
| 7 | 5220.16 |
| 8 | 11282.94 |
| 10 | 2417.69 |
| 12 | 2210.91 |
| 13 | 1987.42 |
| 16 | 4468.86 |
| 19 | 3150.98 |
| 23 | 2708.1 |
| 26 | 2166.93 |
| 34 | 2113.69 |
| 166 | 2485.15 |
### Chart: GM-CSF
| Category | Hu GM-CSF (34) |
|---|---|
| 2 | 52.3 |
| 3 | 54.03 |
| 4 | 72.93 |
| 5 | 89.08 |
| 6 | 72.24 |
| 7 | 63.11 |
| 8 | 65.36 |
| 10 | 53.46 |
| 12 | 47.77 |
| 13 | 47.46 |
| 16 | 49.01 |
| 19 | 46.34 |
| 23 | 48.08 |
| 26 | 39.67 |
| 34 | 44.38 |
| 166 | 32.04 |pg/mL
pg/mL
pg/mL
### Chart: SCF
| Category | Hu SCF (65) |
|---|---|
| 2 | 119.2 |
| 3 | 123.99 |
| 4 | 166.53 |
| 5 | 150.03 |
| 6 | 112.03 |
| 7 | 123.99 |
| 8 | 124.31 |
| 10 | 99.46 |
| 12 | 75.31 |
| 13 | 64.83 |
| 16 | 59.44 |
| 19 | 79.75 |
| 23 | 81.34 |
| 26 | 73.72 |
| 34 | 81.34 |
| 166 | 102.64 |
### Chart: VEGF
| Category | Hu VEGF (45) |
|---|---|
| 2 | 0.0 |
| 3 | 0.0 |
| 4 | 0.0 |
| 5 | 186.14 |
| 6 | 172.32 |
| 7 | 225.63 |
| 8 | 335.51 |
| 10 | 0.0 |
| 12 | 0.0 |
| 13 | 0.0 |
| 16 | 114.01 |
| 19 | 0.0 |
| 23 | 0.0 |
| 26 | 0.0 |
| 34 | 0.0 |
| 166 | 0.0 |
### Chart: NGF-β
| Category | Hu b-NGF (46) |
|---|---|
| 2 | 15.03 |
| 3 | 16.09 |
| 4 | 26.03 |
| 5 | 20.82 |
| 6 | 28.05 |
| 7 | 21.29 |
| 8 | 19.99 |
| 10 | 6.7 |
| 12 | 12.32 |
| 13 | 11.15 |
| 16 | 16.56 |
| 19 | 13.5 |
| 23 | 10.21 |
| 26 | 3.91 |
| 34 | 2.76 |
| 166 | 4.61 |
### Chart: IFN-λ2
| Category | |
|---|---|
| 2 | 321.62070167064667 |
| 3 | 321.1890235543251 |
| 4 | 320.4906784892082 |
| 5 | 602.868793785572 |
| 6 | 666.4939257502556 |
| 7 | 618.0172765254974 |
| 8 | 557.9911229610443 |
| 10 | 443.8608595728874 |
| 12 | 440.2469456791878 |
| 13 | 448.6096083521843 |
| 16 | 454.0677351951599 |
| 19 | 396.80633306503296 |
| 23 | 373.71850472688675 |
| 26 | 357.16026705503464 |
| 34 | 352.1206123828888 |
| 166 | 321.7760623693466 |pg/mL
